# An effective approach for fault diagnosis: Conflict management and BBA generation

**DOI:** 10.1371/journal.pone.0324603

**Published:** 2025-06-05

**Authors:** Yuhao Qin, Zhike Qiu, Zichong Chen, Rui Cai

**Affiliations:** 1 Business College, Southwest University, Chongqing, China; 2 School of Information and Communication Engineering, University of Electronic Science and Technology of China, Chengdu, China; Northwestern Polytechnical University, CHINA

## Abstract

Evidence Theory (ET) is widely applied to handle uncertainty issues in fault diagnosis. However, when dealing with highly conflicting evidence, the use of Dempster’s rule may result in outcomes that contradict reality. To address this issue, this paper proposes a fault diagnosis decision-making method. The method is primarily divided into two parts. First, a similarity measurement method is introduced to solve the conflict management problem. This method combines the belief and plausibility functions within ET. It not only considers the numerical similarity between pieces of evidence but also takes into account directional similarity, better capturing the differences between different pieces of evidence. The effectiveness of this method is validated through several complex numerical examples. Next, based on this measurement method, we propose a conflict management method, which is validated through comparative experiments. Then, considering the inherent uncertainty in real-world sensor data, we propose a basic belief assignment (BBA) generation method based on Student’s t-distribution and fuzzy membership functions. Finally, by combining the proposed conflict management method based on similarity measurement with the BBA generation method, we derive the final fault diagnosis decision, and its effectiveness is demonstrated through an application.

## Introduction

Fault diagnosis technology is widely applied in engineering systems. By detecting, isolating, and identifying faults, it can effectively enhance the safety and reliability of the system. However, for complex engineering systems, reliability analysis and fault diagnosis are highly challenging tasks. As is well-known, a single sensor cannot fully capture enough system state information. For example, imagine we have three sensors detecting the pressure of a device. The first sensor shows 50 psi, the second shows 55 psi, and the third shows 53 psi. Suppose that the device fails if the pressure exceeds 52 psi. The problem lies in how to determine whether the device has failed based on the results from these sensors. Based on the information from these sensors, the result is uncertain, because a single sensor’s data may miss critical information, leading to different judgment results. In practice, data from different sensors may conflict, be uncertain, ambiguous, or even contradictory.

Therefore, how to process this information and make the correct decision is a highly challenging and significant problem. Several theories have emerged to address uncertainty and fuzziness, such as probability theory [1], evidence theory(ET) [2–4], fuzzy set theory [5,6], and neuro-fuzzy set theory [7]. Among these methods, ET has advantages in handling uncertainty, representing an important development in Bayesian probability theory [8]. This theory was originally proposed by Dempster and later improved by Shafer. Due to this advantage, it has been widely applied in fields such as fault diagnosis [9–11], information fusion [12,13], multi-attribute decision analysis [14,15], complex evidence theory [16], decision-making [17,18], and medical image analysis [19].

Although ET has received widespread attention due to its advantages, there are still some limitations that need further research. One significant issue is that when the evidence is highly conflicting, the Dempster’s combination rule can yield results that contradict common sense. As a result, many scholars have developed various methods to address this problem. These methods can be broadly categorized into two types. The first type focuses on improving the combination rule. Many scholars have proposed alternative combination rules, such as Yager’s rule [20] and Dubois and Prade’s rule [21]. Although these methods can effectively address the problems caused by conflict to some extent, they lose some desirable properties of the Dempster’s rule, such as commutativity and associativity. Additionally, these methods often perform poorly in terms of computational efficiency, with their complexity growing exponentially when the quantity of evidence grows. As a result, some scholars have proposed a second solution. The second method involves modifying the evidence before combination. For instance, Deng *et al*. [22] used evidence distance to measure conflicts between pieces of evidence and assess the reliability of each piece. Xiao [23] introduced the Belief Jensen-Shannon divergence, a novel metric used to measure differences between different pieces of evidence. However, Xiao’s approach neglects the role of multiple subsets when measuring conflict. Furthermore, methods based on divergence and similarity [24–28] have also seen some development. Although existing methods have achieved some success, they primarily focus on numerical differences and fail to fully account for the directional consistency of evidence. In many practical applications, the directionality of evidence is crucial for assessing its consistency, especially when conflicts exist between multiple subsets of evidence. Directional similarity measurement, by quantifying the directional consistency of evidence, enhances the consistency measure between pieces of evidence while preserving numerical similarity, making conflict management more precise. When facing high conflict or incomplete evidence, directional similarity helps us better understand the relevance of evidence, avoiding an over-reliance on numerical similarity, thereby improving the reliability and accuracy of decision-making.

In this paper, we introduce a novel fault diagnosis method designed to enhance decision-making accuracy when dealing with uncertainty and conflicting evidence in complex engineering systems. This method consists of two primary components. On one hand, we propose a new similarity measurement approach that accounts for differences between pieces of evidence from both numerical and directional perspectives. Specifically, numerical similarity is assessed using Euclidean distance [29], while directional similarity is quantified through the vector product, which captures the differences in direction and structure between the various pieces of evidence. This approach allows for a more precise measurement of the discrepancies between evidence. We demonstrate the reliability and applicability of the proposed similarity measurement through mathematical proofs and several complex numerical examples. On the other hand, considering that in practical applications, sensor measurements often experience real-time fluctuations that introduce inherent uncertainty into the data, we utilize the Student’s t-distribution [30] and fuzzy membership functions [31] to construct the basic belief assignment (BBA). Finally, we combine the proposed conflict management method based on similarity measurement with the BBA generation method, applying them to fault diagnosis.

The key contributions of this study are as follows:

1. We design a new fault diagnosis method aimed at improving decision-making accuracy when handling uncertainty and conflicting evidence in complex engineering systems.

2. We propose a novel similarity measure that combines numerical similarity and directional similarity. By integrating the proposed similarity measure with Deng entropy and information volume, we obtain a new conflict management method that can effectively address the conflict problem in evidence theory.

3. In order to address the issue of inherent uncertainty in data due to the fluctuations often observed in sensor measurements in real-time, we used a fuzzy membership function based on the Student’s t-distribution to construct basic belief assignment (BBA), and combined it with the proposed conflict management algorithm for fault diagnosis, which yielded promising results.

## Preliminaries

Evidence Theory (ET) [32,33] is a powerful framework designed to handle uncertain information, initially introduced by Dempster and Shafer. It has found widespread application in various fields. The strength of ET lies in its ability to combine information from different sources, even when the available data is incomplete or ambiguous. Evidence in this framework is represented by belief functions and plausibility functions, which quantify the degree of certainty about different hypotheses. The fusion of evidence from multiple sources is accomplished using Dempster’s rule, which resolves conflicts and allows for more reliable decision-making.

**Definition 1** (Framework of discernment). *Let Ω be a collection of N distinct and complete elements. This collection is referred to as the frame of discernment (FOD), and is expressed as:*

$$\Omega =\{\gamma_{1},\gamma_{2},\ldots ,\gamma_{N}\}$$ (1)

*The power set of Ω, denoted $2^{\Omega }$, consists of all possible subsets of Ω, which can be written as:*

$$2^{\Omega }=\{\emptyset ,\{\gamma_{1}\},\{\gamma_{2}\},\ldots ,\{\gamma_{N}\},\{\gamma_{1},\gamma_{2}\},\ldots ,\Omega \}$$ (2)

**Definition 2** (Basic belief assignment). *It is defined as a mapping m from the power set $2^{\Omega }$ to the interval [0,1], subject to the following conditions:*

$$\{\sum_{\gamma \in 2^{\Omega }}m(\gamma )=1\;m(\emptyset )=0$$ (3)

*where m(γ) represents the degree of belief assigned to the subset γ.*

**Definition 3** (Belief and plausibility functions). Bel(γ)* and Pl(γ) are defined as follows:*

$$Bel(\gamma )=\sum_{\alpha \subseteq \gamma }m(\alpha )*$$ (4)

$$Pl(\gamma )=\sum_{\alpha \cap \gamma \neq \emptyset }m(\alpha )*6pt$$ (5)

*where Bel(γ) represents the mass of belief that supports γ with certainty, and Pl(γ) indicates the mass of belief that might support γ with uncertainty.*

**Definition 4** (Dempster’s rule). *Consider m_1_ and m_2_, two independent BBAs. Dempster’s rule for combining these two BBAs is given by:*

$$m(\gamma )=\frac{1}{1-K}\sum_{\alpha \cap \beta =\gamma }m_{1}(\alpha )\cdot m_{2}(\beta )$$ (6)

*where*

$$K=\sum_{\alpha \cap \beta =\emptyset }m_{1}(\alpha )\cdot m_{2}(\beta )$$ (7)

*Here, K quantifies the degree of conflict between m_1_ and m_2_, and it must satisfy the condition 0 < K < 1 for the combination to be valid.*

## Conflict management method based on similarity measurement

### The analysis of problems in Evidence Theory

ET is generally effective in solving decision-making problems that rely on multiple sources of information. However, when there is a conflict among the available evidence, the theory’s fusion rules tend to fail. The following two examples illustrate this issue.

**Example 3.1.** Consider a frame $\Omega =\{\gamma_{1},\gamma_{2},\gamma_{3}\}$, *m*_1_ and *m*_2_ are the following two BBAs.


$$m_{1}:m_{1}(\gamma_{1})=0.5,\;m_{1}(\gamma_{2})=0.3,\;m_{1}(\gamma_{3})=0.2$$

$$m_{2}:m_{2}(\gamma_{1})=0.5,\;m_{2}(\gamma_{2})=0.3,\;m_{2}(\gamma_{3})=0.2$$

Using Eq (7), we can calculate the value of *K* as 0.62. However, we can find no conflict exists between the two pieces of evidence.

**Example 3.2.** Consider a frame $\Omega =\{\gamma_{1},\gamma_{2},\gamma_{3}\}$, *m*_1_, *m*_2_ and *m*_3_ are the following three BBAs.


$$m_{1}:\;m_{1}(\gamma_{1})=0.98,\;m_{1}(\gamma_{2})=0.01,\;m_{1}(\gamma_{3})=0.01$$

$$m_{2}:\;m_{2}(\gamma_{1})=0,\;m_{2}(\gamma_{2})=0.01,\;m_{2}(\gamma_{3})=0.99$$

$$m_{3}:\;m_{3}(\gamma_{1})=0.9,\;m_{3}(\gamma_{2})=0.1,\;m_{3}(\gamma_{3})=0$$

In this example, we find there are two pieces of evidence strongly support the focal element $\gamma_{1}$, one piece of evidence supports the focal element $\gamma_{3}$, and three pieces of evidence support the focal element $\gamma_{2}$, but with very low support. Now, according to Eq (6), we can calculate $m(\gamma_{1})=m(\gamma_{3})=0$ and $m(\gamma_{2})=1$, and the fusion result is completely inconsistent with the actual fact. Moreover, if the conflict between two pieces of evidence tends to infinity, the denominator in the fusion formula will approach 0, leading to a failure of the fusion rule.

### An enhanced sine similarity measurement

To effectively address the conflict issues in ET, many scholars have proposed improved methods, among which similarity-based approaches have been developed. These methods have achieved good results to some extent. However, how to effectively measure the similarities between pieces of evidence remains a challenging task and still requires further improvement. In this section, we calculate the numerical difference between pieces of evidence based on the Euclidean distance and derive the numerical similarity between the pieces of evidence from this difference. To overcome the inadequacy of determining the evidence weight solely based on the value similarity, we propose a metric based on the directional similarity between pieces of evidence. Ultimately, a new similarity measure is defined through both the numerical similarity and directional similarity, which is then applied to subsequent conflict management.

**Definition 5** (The numerical difference based on Euclidean distance). *Suppose m_1_ and m_2_ are two independent BBAs on Ω, The numerical difference between two pieces of evidence based on the Euclidean distance [29] is defined as follows:*

$$D(m_{1},m_{2})=\sqrt{\sum_{\gamma_{i}\in \Omega }{\left[BPl_{m_{1}}(\gamma_{i})-BPl_{m_{2}}(\gamma_{i})\right]}^{2}}$$ (8)

*where*

$$BPl_{m}(\gamma_{i})=\frac{Bel(\gamma_{i})+Pl(\gamma_{i})}{\sum_{\gamma_{j}\in \Omega }Bel(\gamma_{j})+Pl(\gamma_{j})}$$ (9)

**Remark 1.** Compared to previous studies [23,34], we observe that *BPl*_*m*_ transforms the BBA into a probability distribution by considering both Bel and Pl, which effectively assigns belief values across subsets and avoids the issue of overlooking elements in multiple subsets when calculating similarity.

**Definition 6** (The degree of similarity of value). *Suppose m_1_ and m_2_ are two independent BBAs on Ω, the degree of similarity of value between two pieces of evidence is defined as follows:*

$$S_{v}(m_{1},m_{2})=e^{-D(m_{1},m_{2})}.$$ (10)

**Definition 7** (The degree of similarity of direction). *Suppose m_1_ and m_2_ are two independent BBAs on Ω, the degree of similarity of direction between two pieces of evidence is defined as follows:*

$$S_{d}(m_{1},m_{2})=\frac{\sum_{\gamma_{i}\in \Omega }\left[BPl_{m_{1}}(\gamma_{i})\cdot BPl_{m_{2}}(\gamma_{i})\right]}{\sqrt{\sum_{\gamma_{i}\in \Omega }{\left[BPl_{m_{1}}(\gamma_{i})\right]}^{2}}\cdot \sqrt{\sum_{\gamma_{i}\in \Omega }{\left[BPl_{m_{2}}(\gamma_{i})\right]}^{2}}}.$$ (11)

**Definition 8** (The new similarity). *Suppose m_1_ and m_2_ are two independent BBAs on Ω, the new similarity between two pieces of evidence is defined as follows:*

$$S(m_{1},m_{2})=\frac{S_{v}(m_{1},m_{2})+S_{d}(m_{1},m_{2})}{2}$$ (12)

**Property.** The proposed new similarity measure satisfies the following properties:

1. Bounded: $0\leq S(m_{1},m_{2})\leq 1$.
2. Non-degeneracy: $S(m_{1},m_{2})=1$ if and only if $m_{1}=m_{2}$.
3. Symmetry: $S(m_{1},m_{2})=S(m_{2},m_{1})$.

**Proof.** (1) Given two independent BBAs *m*_1_ and *m*_2_ on Ω, we have:


$$S(m_{1},m_{2})=\frac{S_{v}(m_{1},m_{2})+S_{d}(m_{1},m_{2})}{2}$$

To show that $S(m_{1},m_{2})$ is bounded, we will prove that both $S_{v}(m_{1},m_{2})$ and $S_{d}(m_{1},m_{2})$ are bounded.

For $S_{v}(m_{1},m_{2})$, the term $\left(BPl_{m_{1}}(\gamma_{i})-BPl_{m_{2}}(\gamma_{i}\right){)}^{2}$ is bounded by 0 because the square of the difference is always non-negative.


$$0\leq \sum_{\gamma_{i}\in \Omega }{\left(BPl_{m_{1}}(\gamma_{i})-BPl_{m_{2}}(\gamma_{i})\right)}^{2}$$

Therefore, we can conclude that $D(m_{1},m_{2})\geq 0$.


$$D(m_{1},m_{2})=\sqrt{\sum_{\gamma_{i}\in \Omega }{\left[BPl_{m_{1}}(\gamma_{i})-BPl_{m_{2}}(\gamma_{i})\right]}^{2}}\geq 0$$

Based on the properties of the exponential function, we can conclude that:


$$0\leq S_{v}(m_{1},m_{2})=e^{-D(m_{1},m_{2})}\leq 1$$

For $S_{d}(m_{1},m_{2})$, we have:


$$S_{d}(m_{1},m_{2})=\frac{\sum_{\gamma_{i}\in \Omega }|BPl_{m_{1}}(\gamma_{i})\cdot BPl_{m_{2}}(\gamma_{i})|}{\sum_{\gamma_{i}\in \Omega }|BPl_{m_{1}}(\gamma_{i})|\cdot \sum_{\gamma_{i}\in \Omega }|BPl_{m_{2}}(\gamma_{i})|}$$

Since each $BPl_{m_{1}}(\gamma_{i})$ and $BPl_{m_{2}}(\gamma_{i})$ are in [0, 1], the numerator is between 0 and 1.


$$0\leq \sum_{\gamma_{i}\in \Omega }|BPl_{m_{1}}(\gamma_{i})\cdot BPl_{m_{2}}(\gamma_{i})|\leq \sum_{\gamma_{i}\in \Omega }|BPl_{m_{1}}(\gamma_{i})|\cdot \sum_{\gamma_{i}\in \Omega }|BPl_{m_{2}}(\gamma_{i})|$$

This inequality holds because $\left|BPl_{m_{1}}(\gamma_{i})\cdot BPl_{m_{2}}(\gamma_{i})|\leq |BPl_{m_{1}}(\gamma_{i})|\cdot |BPl_{m_{2}}(\gamma_{i})\right|$, as the absolute value of a product is less than or equal to the product of the absolute values.

Next, let’s consider the denominator. Both sums in the denominator, $\sum_{\gamma_{i}\in \Omega }|BPl_{m_{1}}(\gamma_{i})|$ and $\sum_{\gamma_{i}\in \Omega }|BPl_{m_{2}}(\gamma_{i})|$, are non-negative and are greater than or equal to the individual terms $BPl_{m_{1}}(\gamma_{i})$ and $BPl_{m_{2}}(\gamma_{i})$, which are each bounded by 1. Hence, the sum of these terms in the denominator will never be zero, and it will always be greater than or equal to the numerator.

Thus, we conclude:


$$0\leq S_{d}(m_{1},m_{2})\leq 1$$

So we have thus proven that $S(m_{1},m_{2})$ is bounded, with the specific range $0\leq S(m_{1},m_{2})\leq 1$.

**Proof.** (2) Consider two same BBAs *m*_1_ and *m*_2_ on Ω, we have:


$$S(m_{1},m_{2})=\frac{S_{v}(m_{1},m_{2})+S_{d}(m_{1},m_{2})}{2}$$

For $S_{v}(m_{1},m_{2})$, when $m_{1}=m_{2}$, we get:


$$D(m_{1},m_{2})=\sqrt{\sum_{\gamma_{i}\in \Omega }{\left[BPl_{m_{1}}(\gamma_{i})-BPl_{m_{2}}(\gamma_{i})\right]}^{2}}=0$$

$$S_{v}(m_{1},m_{2})=e^{-D(m_{1},m_{2})}=1$$

For $S_{d}(m_{1},m_{2})$, similarly, we have:


$$S_{d}(m_{1},m_{2})=\frac{\sum_{\gamma_{i}\in \Omega }|BPl_{m_{1}}(\gamma_{i})\cdot BPl_{m_{2}}(\gamma_{i})|}{\sum_{\gamma_{i}\in \Omega }|BPl_{m_{1}}(\gamma_{i})|\cdot \sum_{\gamma_{i}\in \Omega }|BPl_{m_{2}}(\gamma_{i})|}=1$$

Thus:


$$S(m_{1},m_{2})=\frac{1+1}{2}=1$$

If $S(m_{1},m_{2})=1$, it follows that $S_{v}(m_{1},m_{2})=1$ and $S_{d}(m_{1},m_{2})=1$, leading to $m_{1}=m_{2}$.

**Proof.** (3) Consider two independent BBAs *m*_1_ and *m*_2_ on Ω, we have:


$$D(m_{1},m_{2})=\sqrt{\sum_{\gamma_{i}\in \Omega }{\left[BPl_{m_{1}}(\gamma_{i})-BPl_{m_{2}}(\gamma_{i})\right]}^{2}}$$

$$D(m_{2},m_{1})=\sqrt{\sum_{\gamma_{i}\in \Omega }{\left[BPl_{m_{2}}(\gamma_{i})-BPl_{m_{1}}(\gamma_{i})\right]}^{2}}$$

Since


$${\left[BPl_{m_{1}}(\gamma_{i})-BPl_{m_{2}}(\gamma_{i})\right]}^{2}={\left[BPl_{m_{2}}(\gamma_{i})-BPl_{m_{1}}(\gamma_{i})\right]}^{2},$$

It follows that


$$D(m_{1},m_{2})=D(m_{2},m_{1}).$$

Now, let’s calculate the similarity measure $S_{v}(m_{1},m_{2})$:


$$S_{v}(m_{1},m_{2})=e^{-D(m_{1},m_{2})}=e^{-D(m_{2},m_{1})}=S_{v}(m_{2},m_{1})$$

Specifically, since multiplication is commutative, we have $BPl_{m_{1}}(\gamma_{i})\cdot BPl_{m_{2}}(\gamma_{i})=BPl_{m_{2}}(\gamma_{i})\cdot BPl_{m_{1}}(\gamma_{i})$, it follows that $S_{d}(m_{1},m_{2})=S_{d}(m_{2},m_{1})$. So $S(m_{1},m_{2})=S(m_{2},m_{1})$. Therefore, we can conclude that $S(m_{1},m_{2})$ is symmetric.

### Some numerical examples

In this section, we test the similarity measure we proposed through some numerical examples.

**Example 3.3.** Suppose that *m*_1_, *m*_2_, and *m*_3_ are three BBAs on $\Omega =\{\gamma_{1},\gamma_{2},\gamma_{3}\}$.


$$m_{1}:m_{1}(\gamma_{1})=0.40,\;m_{1}(\gamma_{2})=0.30,\;m_{1}(\gamma_{1},\gamma_{3})=0.30$$

$$m_{2}:m_{2}(\gamma_{1})=0.20,\;m_{2}(\gamma_{3})=0.60,\;m_{2}(\gamma_{2},\gamma_{3})=0.20$$

$$m_{3}:m_{3}(\gamma_{1})=0.40,\;m_{3}(\gamma_{2})=0.30,\;m_{3}(\gamma_{1},\gamma_{3})=0.30$$

We can calculate *Bel* and *Pl* as follows:


$$\begin{array}{cccc} m_{1}: & Bel_{1}(\gamma_{1})=0.40, & Bel_{1}(\gamma_{2})=0.30, & Bel_{1}(\gamma_{3})=0.00\\ & Pl_{1}(\gamma_{1})=0.70, & Pl_{1}(\gamma_{2})=0.30, & Pl_{1}(\gamma_{3})=0.30\\ m_{2}: & Bel_{2}(\gamma_{1})=0.20, & Bel_{2}(\gamma_{2})=0.00, & Bel_{2}(\gamma_{3})=0.60\\ & Pl_{2}(\gamma_{1})=0.20, & Pl_{2}(\gamma_{2})=0.20, & Pl_{2}(\gamma_{3})=0.80\\ m_{3}: & Bel_{3}(\gamma_{1})=0.40, & Bel_{3}(\gamma_{2})=0.30, & Bel_{3}(\gamma_{3})=0.00\\ & Pl_{3}(\gamma_{1})=0.70, & Pl_{3}(\gamma_{2})=0.30, & Pl_{3}(\gamma_{3})=0.30 \end{array}$$

The proposed similarity measure for *m*_1_, *m*_2_, and *m*_3_ is calculated as follows:


$$m_{1}:\;BPl_{1}(\gamma_{1})=\frac{0.40+0.70}{0.40+0.30+0.00+0.70+0.30+0.30}=0.55$$

$$BPl_{1}(\gamma_{2})=\frac{0.30+0.30}{0.40+0.30+0.00+0.70+0.30+0.30}=0.30$$

$$BPl_{1}(\gamma_{3})=\frac{0.00+0.30}{0.40+0.30+0.00+0.70+0.30+0.30}=0.15$$

$$m_{2}:\;BPl_{2}(\gamma_{1})=\frac{0.20+0.20}{0.20+0.00+0.60+0.20+0.80+0.20}=0.20$$

$$BPl_{2}(\gamma_{2})=\frac{0.00+0.20}{0.20+0.00+0.60+0.20+0.80+0.20}=0.10$$

$$BPl_{2}(\gamma_{3})=\frac{0.60+0.80}{0.20+0.00+0.60+0.20+0.80+0.20}=0.70$$

$$m_{3}:\;BPl_{1}(\gamma_{1})=\frac{0.40+0.70}{0.40+0.30+0.00+0.70+0.30+0.30}=0.55$$

$$BPl_{1}(\gamma_{2})=\frac{0.30+0.30}{0.40+0.30+0.00+0.70+0.30+0.30}=0.30$$

$$BPl_{1}(\gamma_{3})=\frac{0.00+0.30}{0.40+0.30+0.00+0.70+0.30+0.30}=0.15$$

$$D(m_{1},m_{2})=\sqrt{[0.55-0.20{]}^{2}+[0.30-0.10{]}^{2}+[0.15-0.70{]}^{2}}=0.68$$

$$D(m_{1},m_{3})=\sqrt{[0.55-0.55{]}^{2}+[0.30-0.30{]}^{2}+[0.15-0.15{]}^{2}}=0.00$$

$$D(m_{2},m_{3})=\sqrt{[0.55-0.20{]}^{2}+[0.30-0.10{]}^{2}+[0.15-0.70{]}^{2}}=0.68$$

$$\begin{array}{cc} c@\;cS_{v}(m_{1},m_{2})=e^{-0.68}=0.507 & S_{d}(m_{1},m_{2})=\frac{0.245}{0.474}=0.517\\ S_{v}(m_{1},m_{3})=e^{0.00}=1.000 & S_{d}(m_{1},m_{3})=\frac{0.505}{0.505}=1.000\\ S_{v}(m_{2},m_{3})=e^{-0.68}=0.507 & S_{d}(m_{2},m_{3})=\frac{0.245}{0.474}=0.517 \end{array}$$

$$S(m_{1},m_{2})=\frac{0.507+0.517}{2}=0.512$$

$$S(m_{1},m_{3})=\frac{1.000+1.000}{2}=1.000$$

$$S(m_{2},m_{3})=\frac{0.507+0.517}{2}=0.512$$

From the calculation results, we can observe that $S(m_{1},m_{2})=S(m_{2},m_{3})<S(m_{1},m_{3})=1$, which aligns with human intuition.

**Example 3.4.** Assume that $\Omega =\{\gamma_{1},\ldots ,\gamma_{14}\}$, and the BBAs are as follows:


$$m_{1}:\;m_{1}(\gamma_{2})=\lambda ,\;m_{1}(N)=1-\lambda$$

$$m_{2}:\;m_{2}(\gamma_{2})=0.99,\;m_{2}(N)=0.01$$

**Fig 1:**
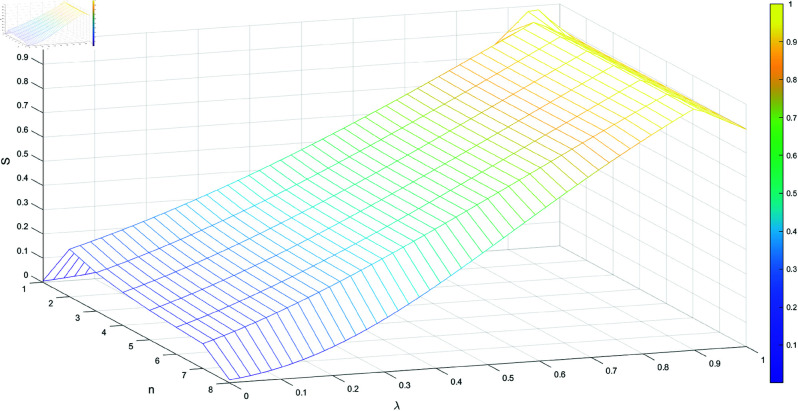
S varying with t and |F|.

**Fig 2:**
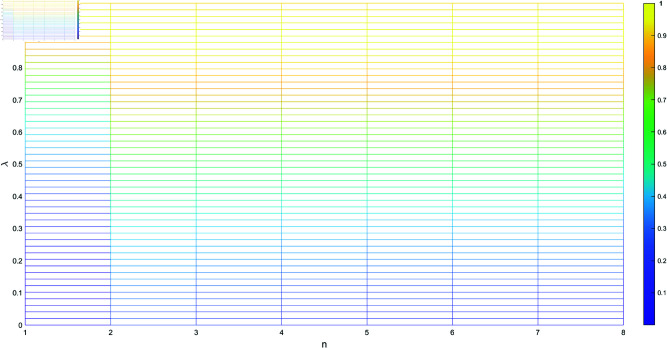
The values of n and λ.

**Fig 3:**
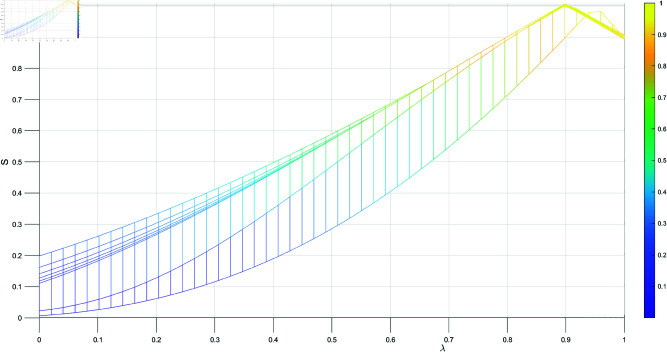
The similarity varying with λ.

**Fig 4:**
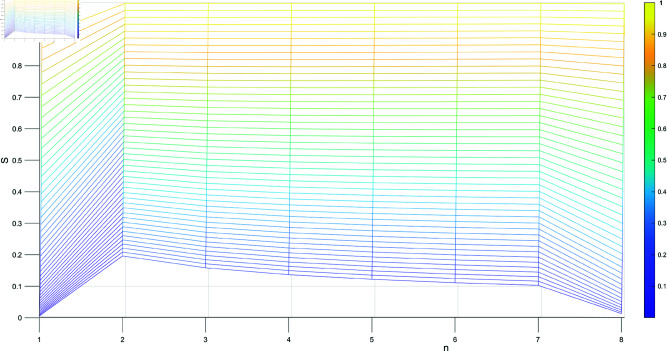
The similarity varying with n.

Table 1 shows the trend of the subset *N*, *n* represents the different combinations of the subset *N*, starting from the subset with the fewest elements and ending with the subset containing the most elements, where the parameter λ varies from 0 to 1. As illustrated in Figs 1, 2, 3, and 4, when λ=0.99, we have $m_{1}=m_{2}$, and the sine similarity measure reaches the maximum value of 1. The sine similarity measure always stays between 0 and 1, as confirmed by this example.

This example demonstrates the effectiveness of the new similarity measure we proposed.

**Table 1:** Different values for subset X.

n	N
1	$\left\{\gamma_{1}\right\}$
2	$\left\{\gamma_{1},\gamma_{2}\right\}$
3	$\left\{\gamma_{1},\gamma_{2},\gamma_{3}\right\}$
4	$\left\{\gamma_{1},\gamma_{2},\gamma_{3},\gamma_{4}\right\}$
5	$\left\{\gamma_{1},\gamma_{2},\gamma_{3},\gamma_{4},\gamma_{5}\right\}$
6	$\left\{\gamma_{1},\gamma_{2},\gamma_{3},\gamma_{4},\gamma_{5},\gamma_{6}\right\}$
7	$\left\{\gamma_{1},\gamma_{2},\gamma_{3},\gamma_{4},\gamma_{5},\gamma_{6},\gamma_{7}\right\}$
8	$\left\{\gamma_{1},\gamma_{2},\gamma_{3},\gamma_{4},\gamma_{5},\gamma_{6},\gamma_{7},\gamma_{8}\right\}$

Table notes: This table shows the values of subsets X for different values of n.

### A novel conflict management method

In this section, we propose a new conflict management method that utilizes the newly defined similarity measure to assess the degree of conflict between pieces of evidence and employs Deng entropy [35] to measure the uncertainty among them. Based on these metrics, we determine the weights to adjust each piece of evidence accordingly. Finally, we apply Dempster’s rule to obtain the final result. The specific steps of the proposed method are as follows:

**Step 1:** Utilize Eq (12) to determine the similarity between two pieces of evidence, *m*_*j*_ and *m*_*k*_. Then the similarity measure matrix ${\text{SMM}}_{n\times n}$ can be represented as follows:

$${\text{SMM}}_{n\times n}=[\begin{array}{cccc} 1 & S(m_{1},m_{2}) & \cdots & S(m_{1},m_{n})\\ S(m_{2},m_{1}) & 1 & \cdots & S(m_{2},m_{n})\\ \vdots & \vdots & \ddots & \vdots \\ S(m_{n},m_{1}) & S(m_{n},m_{2}) & \cdots & 1 \end{array}]$$ (13)

**Step 2:** Compute the support degree using the following formula:

$$\text{Sup}(m_{j})=\sum_{\begin{array}{c} k=1\\ k\neq j \end{array}}^{n}S(m_{j},m_{k})$$ (14)

**Step 3:** Compute the uncertainty based on Deng entropy as follows:

$$\text{uncer}(m_{j})=\frac{E_{d}(m_{j})}{\sum_{i}E_{d}(m_{j})}$$ (15)

where

$$E_{d}(m_{j})=-\sum_{\gamma_{i}\in \Omega }BPl(\gamma_{i})\log_{2}\frac{BPl(\gamma_{i})}{2^{\left|\gamma_{i}\right|}-1}$$ (16)

**Step 4:** Compute the information volume as follows:

$$IV(m_{j})=e^{\text{uncer}(m_{j})}$$ (17)

**Step 5:** Compute the the weight of *m*_*j*_ based on *Sup* and *IV* as follows:

$$w(m_{j})=\frac{Sup(m_{j})\times IV(m_{j})}{\sum_{j=1}^{n}Sup(m_{j})\times IV(m_{j})}$$ (18)

**Step 6:** Compute the adjusted average evidence using the following equation:

$$m^{.}(\gamma )=\sum_{j=1}^{n}w_{j}*m_{j}(\gamma )$$ (19)

**Step 7:** Apply Dempster’s rule iteratively for *n*–1 times on the adjusted average evidence to derive the final outcome:

$$m_{⊕}(\gamma )=(m^{.}(\gamma )⊕m^{.}(\gamma ))⊕\ldots ⊕m^{.}(\gamma {)}_{n-1}$$ (20)

The proposed method is shown in Algorithm 1.

**Algorithm 1. Conflict management method.**



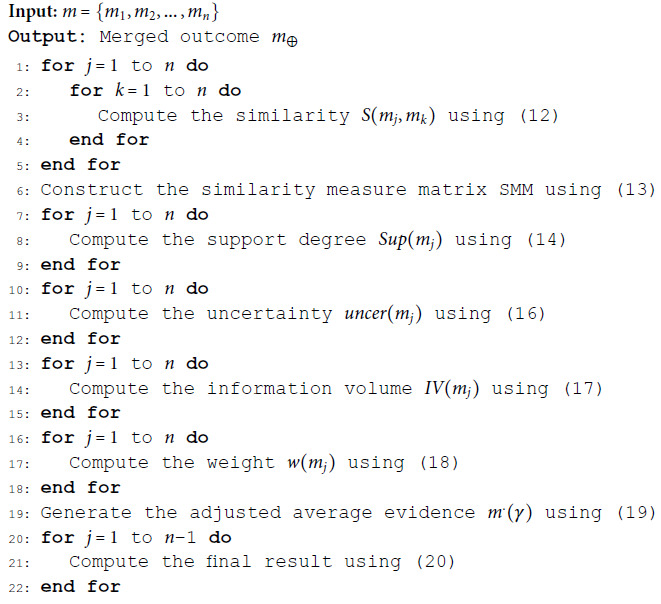



### Experiment for proposed conflict management method

In this section, we validate the effectiveness of the conflict management method we proposed through comparative experiments.

Assume that $\Omega =\{\gamma_{1},\gamma_{2},\gamma_{3}\}$. We have five different pieces of evidence, which are shown in Table 2. These pieces of evidence come from five distinct sensors. Upon examination, we observe that the second piece of evidence contains a high level of uncertainty, while the fifth piece of evidence is significantly inconsistent with the rest.

The specific calculation process is as follows:

**Table 2:** Five BBAs.

BBAs	$\left\{\gamma_{1}\right\}$	$\left\{\gamma_{2}\right\}$	$\left\{\gamma_{3}\right\}$	$\left\{\gamma_{1},\gamma_{2}\right\}$	$\left\{\gamma_{2},\gamma_{3}\right\}$	Ω
$m_{1}^{*}$	0.60	0.08	0.08	0.08	0.04	0.12
$m_{2}^{*}$	0.16	0.24	0.45	0.00	0.00	0.15
$m_{3}^{*}$	0.80	0.00	0.05	0.00	0.00	0.15
$m_{4}^{*}$	0.52	0.08	0.08	0.00	0.08	0.24
$m_{5}^{*}$	0.00	0.25	0.45	0.00	0.15	0.15

Table notes: This table shows five BBAs with their respective values for various subsets.

**Step 1.** Using the Eq (13), we can calculate the similarity measure matrix as follows:

Here is the LaTeX code for the matrix in the image:


$$SSM=[\begin{array}{ccccc} 1 & 0.5647 & 0.7758 & 0.9124 & 0.2602\\ 0.5647 & 1 & 0.3983 & 0.6023 & 0.2669\\ 0.7758 & 0.3983 & 1 & 0.6751 & 0.1209\\ 0.9124 & 0.6023 & 0.6751 & 1 & 0.3321\\ 0.2602 & 0.2669 & 0.1209 & 0.3321 & 1 \end{array}]$$

**Step 2.** So the support degree can be calculated based on Eq (4):


$$\text{Sup}(m_{i}^{*})=\{2.5131,1.8322,1.9701,2.5219,0.9801\}$$

**Step 3.** Then we calculate the uncertainty using Eq (16) as follows:


$$\text{uncer}(m_{i}^{*})=\{2.0432,2.1567,1.6989,2.5345,1.5876\}$$

**Step 4.** So we can get the information volume based on Eq (17):


$$IV(m_{i}^{*})=\{7.7152,8.6426,5.4679,12.6101,4.8920\}$$

**Step 5.** The weight can be calculated using Eq (18):


$$W(m_{i}^{*})=\{0.2348,0.1917,0.1304,0.3850,0.0581\}$$

**Step 6.** So using Eq (19), we can get the final fusion results as follows:


$$m(\gamma_{1})=0.4125$$

$$m(\gamma_{2})=0.1378$$

$$m(\gamma_{3})=0.0912$$

$$m(\gamma_{1},\gamma_{2})=0.1415$$

$$m(\gamma_{2},\gamma_{3})=0.0126$$

m(Ω)=0.2044

**Step 7.** Using Eq (20) for 4 times, we can obtain the final fusion results which are shown as Table 3.

**Table 3:** Different methods for combination results.

BBAs	Method	$\left\{\gamma_{1}\right\}$	$\left\{\gamma_{2}\right\}$	$\left\{\gamma_{3}\right\}$	$\left\{\gamma_{1},\gamma_{2}\right\}$	$\left\{\gamma_{2},\gamma_{3}\right\}$	Ω
*m*_1,2_	Dempster’s rule	0.5498	0.2584	0.0143	0.1501	0.0072	0.0201
	Deng *et al*. [22]	0.5098	0.2738	0.0226	0.1643	0.0094	0.0201
	Xiao [23]	0.5065	0.2689	0.0251	0.1698	0.0095	0.0202
	Li and Xiao [36]	0.4812	0.2954	0.0192	0.1781	0.0080	0.0181
	Gao and Xiao [24]	0.5095	0.2802	0.0201	0.1605	0.0204	0.0204
	Proposed method	0.4972	0.2841	0.0223	0.1678	0.0083	0.0203
*m*_1,2,3_	Dempster’s rule	0.6892	0.2035	0.0124	0.0837	0.0033	0.0079
	Deng *et al*. [22]	0.6633	0.2138	0.0232	0.0842	0.0045	0.0109
	Xiao [23]	0.7136	0.1707	0.0403	0.0648	0.0055	0.0152
	Li and Xiao [36]	0.6582	0.2118	0.0248	0.0877	0.0025	0.0140
	Gao and Xiao [24]	0.7593	0.1599	0.0183	0.0515	0.0057	0.0052
	Proposed method	0.7942	0.1283	0.0142	0.0528	0.0045	0.0059
*m*_1,2,3,4_	Dempster’s rule	0.9074	0.0577	0.0301	0.0027	0.0005	0.0016
	Deng *et al*. [22]	0.8892	0.0735	0.0088	0.0245	0.0010	0.0040
	Xiao [23]	0.8516	0.1016	0.0146	0.0277	0.0015	0.0029
	Li and Xiao [36]	0.8178	0.1301	0.0105	0.0365	0.0012	0.0040
	Gao and Xiao [24]	0.8569	0.0846	0.0180	0.0292	0.0030	0.0042
	Proposed method	0.8587	0.0915	0.0144	0.0295	0.0016	0.0045
*m*_1,2,3,4,5_	Dempster’s rule	0.6379	0.3021	0.0405	0.0145	0.0035	0.0016
	Deng *et al*. [22]	0.8563	0.0285	0.0973	0.0155	0.0011	0.0014
	Xiao [23]	0.8542	0.1117	0.0145	0.0169	0.0008	0.0018
	Li and Xiao [36]	0.8133	0.1504	0.0144	0.0199	0.0007	0.0013
	Gao and Xiao [24]	0.8588	0.1091	0.0113	0.0185	0.0008	0.0015
	Proposed method	0.8753	0.0947	0.0123	0.0155	0.0007	0.0015

The results of different methods are presented in Table 3. Although all methods successfully identify the correct target, they differ in how they assign belief values. When there is little conflict among the evidence, the proposed approach delivers results akin to Dempster’s rule. However, the critical difference arises when an additional piece of evidence, conflicting with existing ones, is introduced: the proposed method maintains a higher belief value under these conditions, unlike Dempster’s rule, which shows a notable decline in belief. Methods that prioritize reliable evidence, such as those by Deng *et al*. [22], use weighted averages. Unfortunately, these methods only address the conflict between evidence, which may not be sufficient in practice. Although methods like Xiao’s [23] and those of Li *et al*. [36] and Gao *et al*. [24] can identify the correct target, they do not perform well in resolving evidence conflicts. In contrast, the proposed approach provides a more comprehensive solution by effectively measuring conflicts between pieces of evidence through the SMM metric and addressing uncertainty with Deng entropy. This enables the proposed method to manage conflicts more efficiently while maintaining high recognition accuracy.

**Fig 5:**
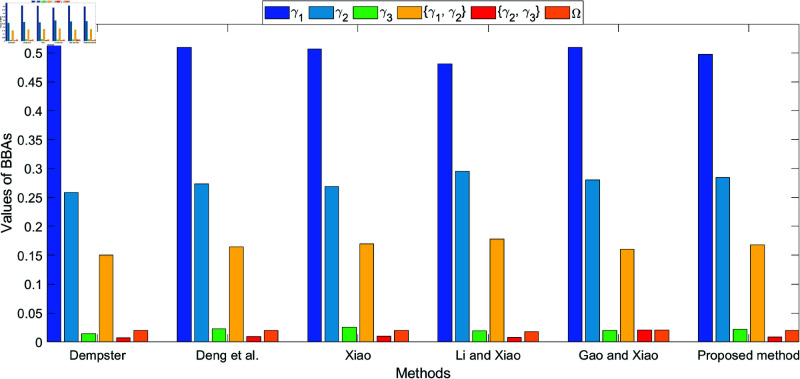
Different method for fusion results at level 1.

**Fig 6:**
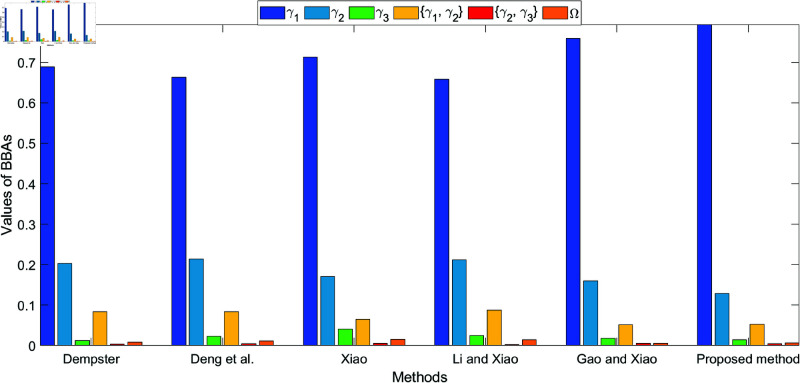
Different method for fusion results at level 2.

**Fig 7:**
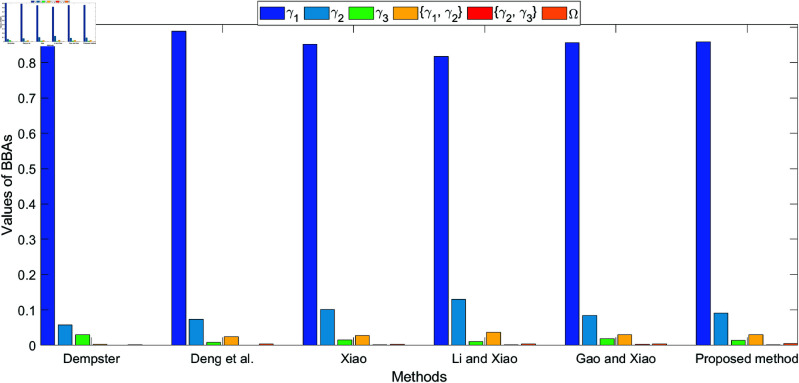
Different method for fusion results at level 3.

**Fig 8:**
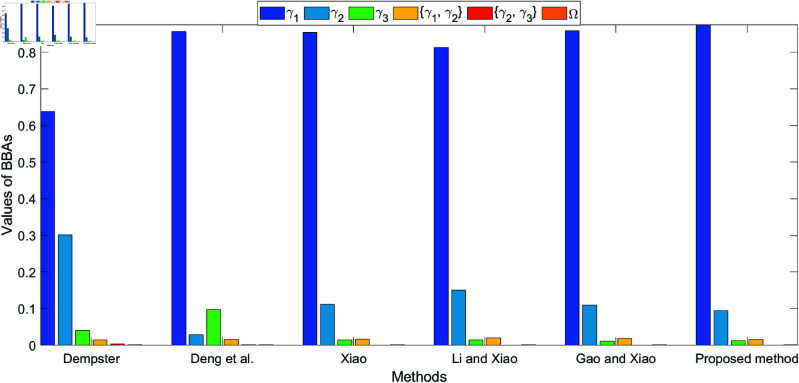
Different method for fusion results at level 4.

The bar plots shown in Figs 5, 6, 7, and 8, which illustrate the fusion results at different levels (level 1 to level 4) using various methods. The figures clearly highlight how the belief values are distributed among the different hypotheses under each method. As conflict levels increase, the proposed method performs consistently better than others by preserving more belief in the correct hypotheses, especially when conflicting evidence is introduced. This performance is demonstrated in the four different levels of evidence conflict, and the proposed method consistently maintains a higher degree of belief in the correct target compared to other methods, even in challenging situations.

## The proposed BBA generation method

Sensor measurements often fluctuate in real-time, causing inherent uncertainty in the data. To manage this, a fuzzy affiliation function [31] is used to create the BBA. The Student’s t-Distribution [30] is chosen because it handles outliers and heavy-tailed data effectively. Unlike the Gaussian distribution [9], the t-Distribution can accommodate larger deviations and has heavier tails, making it more appropriate for environments with noise or extreme values. This distribution helps the affiliation function capture the likelihood that the sample belongs to the t-Distribution, preserving its advantages for real-world sensor data. As a result, we propose a method using the Student’s t-Distribution Affiliation Function to derive the BBA.

The raw dataset from multiple sensors is split into training and testing sets. In the training set, the Student’s t-Distribution Affiliation Function is calculated for each attribute to create models. Testing samples are matched with these models, and the match values are normalized to form BBA functions.

Assuming that the original dataset contains *n* categories, forming the recognition framework $\Theta =\{T_{1},T_{2},\ldots ,T_{n}\}$, with each category consisting of *k* attributes.

(1) Select *m* samples as training data to build models based on attribute distribution. The remaining samples are used for testing, matching them with the pre-built models to calculate the BBA values.

(2) During data preparation, Student’s t-Distribution affiliation functions are used to create models for the training samples across different attributes. This technique improves the model’s precision and reliability by accurately capturing the variation in feature values, thereby offering a more precise representation of the feature range. The membership function for Student’s t-Distribution is as follows:

$$u_{i}(x)={\left(1+\frac{(x-X_{ij}{)}^{2}}{\nu \sigma_{ij}^{2}}\right)}^{-\frac{\nu +1}{2}}$$ (21)

The mean *X*_*ij*_ and sample standard deviation $\sigma_{ij}$ for each category *i* across attributes *j* are computed as:

$$X_{ij}=\frac{1}{q}\sum_{l=1}^{q}x_{ij}^{l}$$ (22)

$$\sigma_{ij}=\sqrt{\frac{1}{q-1}\sum_{l=1}^{q}(x_{ij}^{l}-X_{ij}{)}^{2}}$$ (23)

where i=1,2,…,n;j=1,2,…,k; and $x_{ij}^{l}$ is the value of the *l*-th training sample in category *i* on the *j*-th attribute.

(3)The test samples are compared with the Student’s t-Distribution models to calculate the similarity between the samples and categories. The resulting matching values are normalized to obtain the BBA for each sample. The formula for matching samples with *Q* [9] is:

$$H(Q\leftarrow t)=u_{Q}(X){|}_{x=t}$$ (24)

The test sample’s value for a specific attribute, denoted as *t*, is compared with the Student’s t-Distribution model to assess the match, represented by H(Q←t). This value indicates the degree to which the test sample aligns with the proposition *Q*. After the matching process, the BBA is obtained by ranking the matching values for each category, $H_{1},H_{2},\ldots ,H_{n}$, in descending order, and then computing the BBA accordingly.

$$m_{1,2,...,n}=\frac{H_{n}}{\sum_{i=1}^{n}H_{i}}$$ (25)

(4) Pignistic Probability Function: In cases with multi-subset focal elements in the BBA, the result may indicate multiple focal elements, which can reduce fault identification accuracy. To address this, we apply the pignistic probability transformation [37] to convert the multi-subset BBA into a probability distribution for single-subset elements, improving fault identification. The transformation is defined as:

$$BtPm(T_{i})=\sum_{T\in \Theta }\frac{\left|T_{i}\cap T\right|}{\left|T\right|}m(T)\left(1-m(\emptyset )\right)$$ (26)

where *T* is the identification frame Θ, $\left|T_{i}\cap T\right|$ represents the size of the intersection of *T*_*i*_ and *T*, and *m*(*T*) denotes the BBA value of set *T*.

## Application

**Fig 9:**
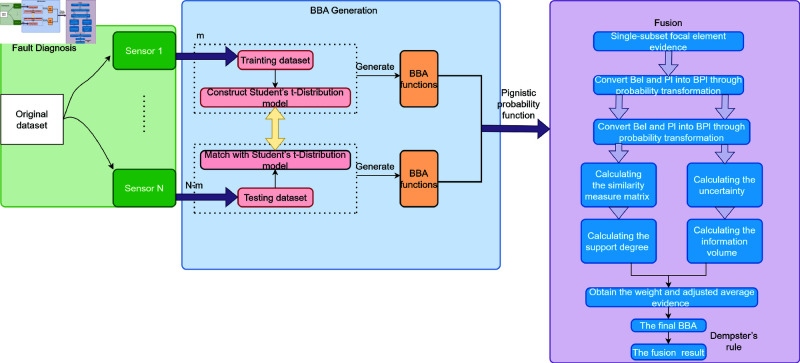
Flowchart of the fault diagnosis method.

In this section, we combine the proposed BBA generation method with conflict management techniques to form a new fault diagnosis method, which shown in Fig 9. It presents the algorithm flow of the fault diagnosis method. The process begins by generating the BBA for each sample from sensor data. Then, it calculates the similarity between different pieces of evidence to manage conflicts. Based on the similarity matrix, the support degree and uncertainty of each piece of evidence are calculated, with uncertainty assessed using Deng entropy. Next, the information volume and weights are computed, adjusting the influence of each piece of evidence. Finally, the adjusted evidence is fused iteratively using Dempster’s rule to obtain the final fault diagnosis result. This approach integrates BBA generation with conflict management, ensuring accurate fault diagnosis even in the presence of conflicting and uncertain evidence.

We selected a case for analysis and validation. The data comes from the open Iris flower dataset from UCI [9]. To highlight the superiority of the method we proposed, we compared it with Xiao’s method [38] and Liu’s method [9]. Xiao proposed a novel BPA generation and fusion method based on cosine similarity and belief entropy. This method first calculates the similarity between the test sample and each focal element within the frame, and then uses Mahalanobis distance and cosine similarity to evaluate the reliability of each BPA. Liu’s method uses the Gaussian affiliation function to convert the raw information collected by sensors into BBA, and then performs evidence weighted fusion based on Bray-Curtis dissimilarity and belief entropy.

There are three types of iris flowers: Setosa (S), Versicolor (E), and Virginica (V), forming the recognition set Θ={S,E,V}, each containing 50 samples, for a total of 150 samples. Each sample is characterized by four features: sepal length (SL), sepal width (SW), petal length (PL), and petal width (PW).

For each iris category (S, E, and V), 30 samples are randomly chosen for training, and the remaining 20 are used for testing. The mean and standard deviation of the 30 training samples across the four features (SL, SW, PL, PW) are computed using Eq (21). The results are shown in Table 4.

**Table 4:** Training samples’ mean and standard deviation.

Category	$\langle X_{SL},\sigma_{SL}\rangle$	$\langle X_{SW},\sigma_{SW}\rangle$	$\langle X_{PL},\sigma_{PL}\rangle$	$\langle X_{PW},\sigma_{PW}\rangle$
S	(5.1023, 0.3649)	(3.4900, 0.3411)	(1.4712, 0.1814)	(0.2465, 0.0992)
E	(6.0732, 0.5365)	(2.7910, 0.3225)	(4.3321, 0.4511)	(1.3546, 0.2078)
V	(6.5907, 0.6782)	(2.9413, 0.3295)	(5.6041, 0.6163)	(2.0071, 0.2517)

**Fig 10:**
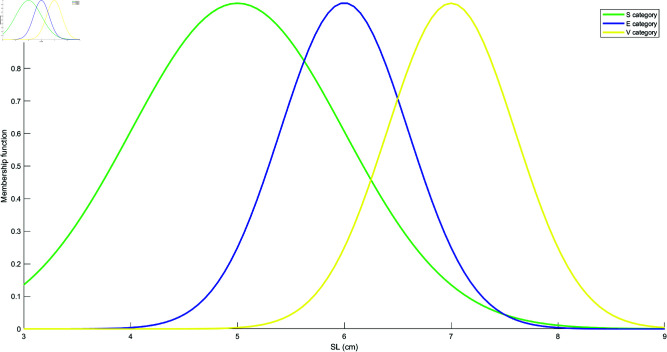
Student’s t-Distribution model for attribute SL.

**Fig 11:**
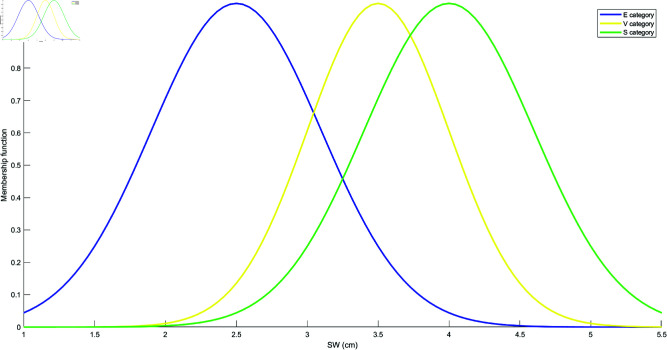
Student’s t-Distribution model for attribute SW.

**Fig 12:**
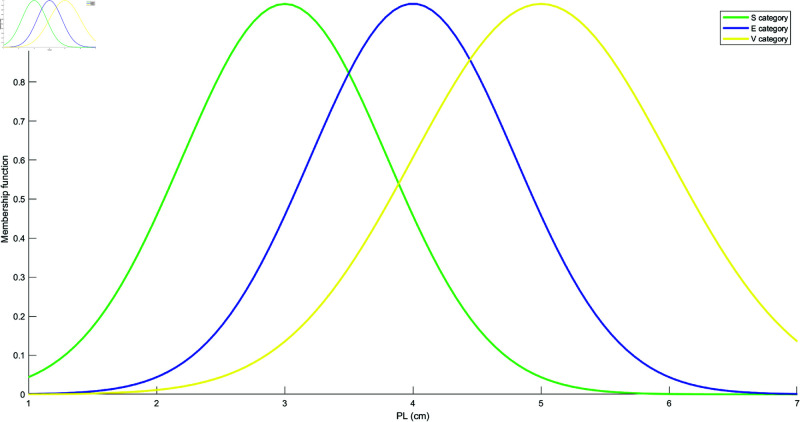
Student’s t-Distribution model for attribute PL.

**Fig 13:**
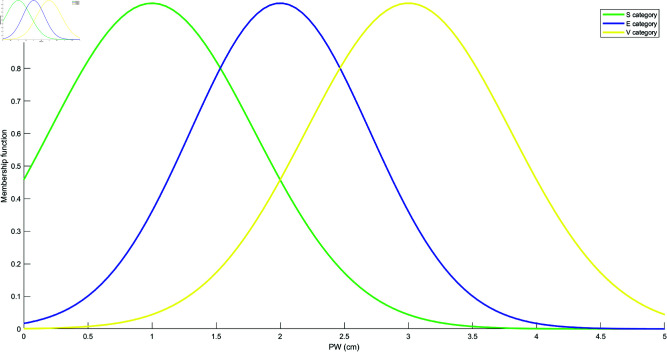
Student’s t-Distribution model for attribute PW.

Based on the mean and standard deviation of the training samples in each category, the models for each attribute are constructed using the Student’s t-Distribution for each category. The resulting models for the four attributes are shown in Figs 10, 11, 12, and 13. These models are generated by fitting the Student’s t-Distribution to the data, reflecting the distinct nature of each category’s distribution on the respective attributes.

For the SL attribute, the membership functions $u_{S}(\gamma )$, $u_{E}(\gamma )$, and $u_{V}(\gamma )$ represent S, E, and V as the categories. The $u_{SE}(\gamma )$ shows that the attribute can belong to both categories S and E. Similarly, $u_{SEV}(\gamma )$ reflects the classification of the attribute into S, E, and V categories. The equations for these membership functions are as follows:

$$u_{SE}(\gamma )=\min(u_{S}(\gamma ),u_{E}(\gamma ))$$ (27)

$$u_{SEV}(\gamma )=\min(u_{S}(\gamma ),u_{E}(\gamma ),u_{V}(\gamma ))$$ (28)

Then, by matching the test samples with the Student’s t-distribution models, we calculate the degree of match between the test sample and the corresponding category. After normalization, the BBA is obtained.

**Fig 14:**
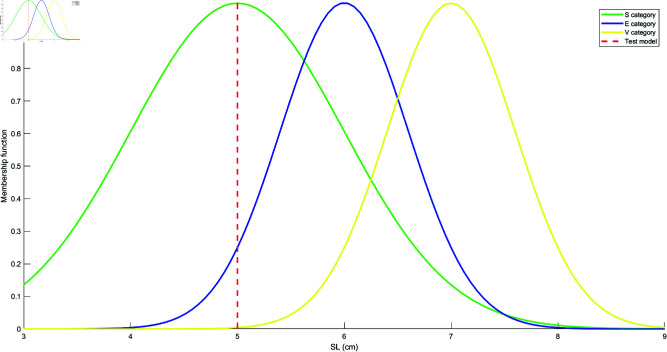
Matching degree between testing sample and Gaussian models for SL.

**Fig 15:**
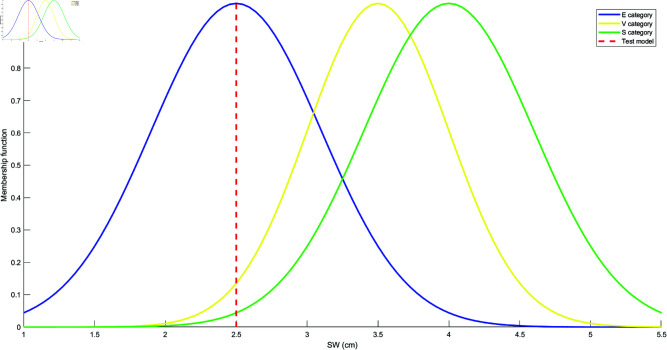
Matching degree between testing sample and Gaussian models for SW.

**Fig 16:**
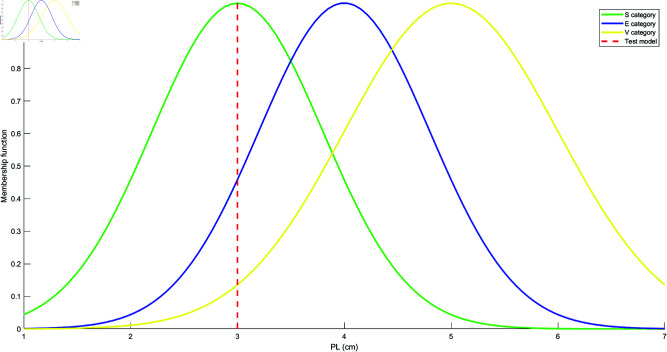
Matching degree between testing sample and Gaussian models for PL.

**Fig 17:**
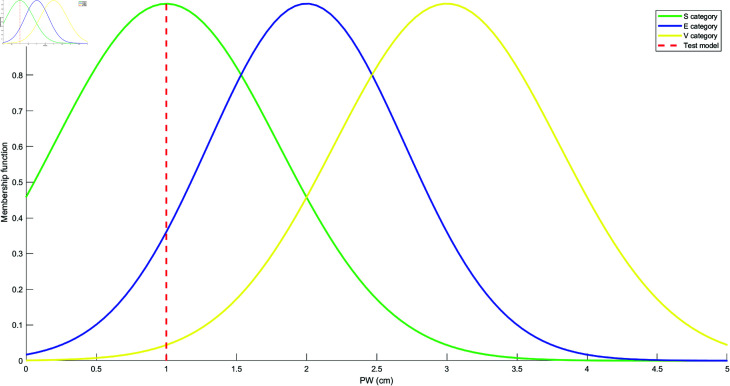
Matching degree between testing sample and Gaussian models for PW.

For instance, to evaluate class S, we randomly select a sample $\gamma =[\gamma_{1},\gamma_{2},\gamma_{3},\gamma_{4}]=[4.2,3.4,1.2,0.3]$, where $\gamma_{1},\gamma_{2},\gamma_{3},\gamma_{4}$ represent the feature values of the test sample for SL, SW, PL, and PW, respectively. This sample is matched with the Student’s t-distribution models, and the degree of match is computed, as shown in Figs 14, 15, 16, and 17.

Four sets of BBA values for the test sample were calculated using the attributes SL, SW, PL, and PW. The numerical results are shown in Table 5.

**Table 5:** BBA values for the test sample across attributes.

Category	m({S})	m({E})	m({V})	m({S,E})	m({S,V})	m({E,V})
$m_{1}(BPA_{SL})$	0.9432	0.0000	0.0000	0.2284	0.0000	0.0000
$m_{2}(BPA_{SW})$	0.0000	0.0000	0.9612	0.0000	0.0000	0.8633
$m_{3}(BPA_{PL})$	0.9657	0.0000	0.0000	0.0000	0.0000	0.0000
$m_{4}(BPA_{PW})$	0.9913	0.0000	0.0000	0.0456	0.0000	0.0000

The BPA functions have high conflict due to multiple subset focal elements, making direct fusion unreliable. To address this, they are converted into single-subset focal elements through pignistic probability transformation. The results are in Table 6. At the same time, the BBA results obtained using Xiao’s method and Liu’s method are shown in Tables 7 and 8.

**Table 6:** The final BBAs for proposed method.

Category	m({S})	m({E})	m({V})
$m_{1}^{*}(BPA_{SL})$	0.8654	0.1158	0.0188
$m_{2}^{*}(BPA_{SW})$	0.1096	0.2624	0.6280
$m_{3}^{*}(BPA_{PL})$	0.9996	0.0002	0.0002
$m_{4}^{*}(BPA_{PW})$	0.9802	0.0197	0.0001

**Table 7:** The final BBAs for Xiao’s method.

Category	m({S})	m({E})	m({V})
$m_{1}^{*}(BPA_{SL})$	0.8623	0.1130	0.0247
$m_{2}^{*}(BPA_{SW})$	0.1098	0.2644	0.6258
$m_{3}^{*}(BPA_{PL})$	0.9994	0.0003	0.0003
$m_{4}^{*}(BPA_{PW})$	0.9756	0.0223	0.0021

**Table 8:** The final BBAs for Liu’s method.

Category	m({S})	m({E})	m({V})
$m_{1}^{*}(BPA_{SL})$	0.8601	0.1145	0.0254
$m_{2}^{*}(BPA_{SW})$	0.1052	0.2668	0.6280
$m_{3}^{*}(BPA_{PL})$	0.9998	0.0001	0.0001
$m_{4}^{*}(BPA_{PW})$	0.9798	0.0201	0.0001

Using the Eq (13), we can calculate the similarity measure matrix as follows:


$$SMM=[\begin{array}{cccc} 1 & 0.1154 & 0.8423 & 0.9123\\ 0.1154 & 1 & 0.0145 & 0.0097\\ 0.7945 & 0.0145 & 1 & 0.9542\\ 0.8423 & 0.0097 & 0.9542 & 1 \end{array}]$$

So the support degree can be calculated based on Eq (14):


$$\text{Sup}(m_{i}^{*})=\{0.8062,0.1396,1.7632,1.8699\}$$

Then we calculate the uncertainty using Eq (16) as follows:


$$\text{uncer}(m_{i}^{*})=\{2.0145,0.9854,1.6534,2.0431\}$$

So we can get the information volume based on Eq (17):


$$IV(m_{i}^{*})=\{7.4970,2.6789,5.2248,7.7145\}$$

The weight can be calculated using Eq (18):


$$W(m_{i}^{*})=\{0.2011,0.0125,0.3065,0.4799\}$$

So using Eqs (19) and (20), we can get the final results are shown in Table 9.

**Table 9:** Final fusion results for different method.

BBAs	Method	m({S})	m({E})	m({V})
$m_{1}^{*}⊕m_{2}^{*}$	Xiao’s method [38]	0.9756	0.0189	0.0055
	Liu’s method [9]	0.9821	0.0142	0.0037
	Proposed method	0.9952	0.0022	0.0026
$m_{1}^{*}⊕m_{2}^{*}⊕m_{3}^{*}$	Xiao’s method [38]	0.9806	0.0097	0.0097
	Liu’s method [9]	0.9886	0.0057	0.0057
	Proposed method	0.9998	0.0001	0.0001
$m_{1}^{*}⊕m_{2}^{*}⊕m_{3}^{*}⊕m_{4}^{*}$	Xiao’s method [38]	0.9842	0.0029	0.0029
	Liu’s method [9]	0.9904	0.0048	0.0048
	Proposed method	1.0000	0.0000	0.0000

Table notes: This table shows the final fusion results for different methods across various BBAs.

From the table results, it can be seen that all three methods successfully identified the sample category as category S with confidence levels of 1.0000, 0.9842, and 0.9904, respectively. However, the method we proposed achieved the highest confidence level, demonstrating the effectiveness and superiority of our method.

The remaining 59 training samples were tested, and Xiao’s method and Liu’s method achieved overall recognition rates of 97.24% and 97.68%, respectively, while our method achieved an overall recognition rate of 98.75%.

## Conclusion

This paper proposes a new fault diagnosis decision-making method, which consists of two main components. On the one hand, to address the limitations of existing similarity measurement methods, we introduce an innovative similarity measurement that combines the belief and plausibility functions in evidence theory. This method not only considers the numerical similarity between pieces of evidence but also takes into account the directional similarity, better capturing the differences between different pieces of evidence. Through several complex numerical examples, we demonstrate that this measurement satisfies several ideal properties. Based on this measurement, we then propose a new conflict management method. To verify its effectiveness, we conducted experiments using five different sources of evidence. Compared with other methods, our approach not only successfully identifies the correct target but also maintains a higher belief value. On the other hand, considering the inherent uncertainty in real-world sensor data, we propose a new basic belief assignment (BBA) generation method, which utilizes Student’s t-distribution and fuzzy membership functions to generate the new BBA. Finally, we combine the conflict management method based on the new similarity measurement with the new BBA generation method, resulting in the final fault diagnosis decision-making method. We selected the publicly available Iris flower dataset for case analysis and compared it with existing methods by Xiao and Liu. The experimental results demonstrate the effectiveness of our approach, showing higher diagnostic accuracy, especially when handling evidence with conflicts and uncertainties. However, the proposed method still has some limitations, such as potentially poor computational efficiency. In the future, we will optimize and improve this method, for instance, by using heuristic algorithms [39] to enhance our approach and eventually apply it to other fields involving conflict management.

## Supporting information

Data availability (DOCX)
