# Supplementary material for: An effective approach for fault diagnosis: Conflict management and BBA generation
Source: PLoS One. 2025 Jun 5;20(6):e0324603. doi: 10.1371/journal.pone.0324603 (PMC12140396; doi:10.1371/journal.pone.0324603)
Supplement: Data availability — (DOCX) [file pone.0324603.s001.docx]

In the application section (Section 5) of our manuscript, we used the Iris dataset from the UCI Machine Learning Repository, which can be accessed at the following link: <http://archive.ics.uci.edu/ml/datasets/iris>. We have also cited this dataset in the relevant section of the article.
